# Population Risk Improvement with Model Compression: An Information-Theoretic Approach †

**DOI:** 10.3390/e23101255

**Published:** 2021-09-27

**Authors:** Yuheng Bu, Weihao Gao, Shaofeng Zou, Venugopal V. Veeravalli

**Affiliations:** 1 Department of Electrical and Computer Engineering, University of Illinois at Urbana-Champaign, Urbana, IL 61820, USA; buyuheng@mit.edu; 2 Bytedance Inc., Bellevue, WA 98004, USA; weihao.gao@bytedance.com; 3 Department of Electrical Engineering, University at Buffalo, The State University of New York, Buffalo, NY 14221, USA; szou3@buffalo.edu

**Keywords:** empirical risk, generalization error, K-means clustering, model compression, population risk, rate distortion theory, vector quantization

## Abstract

It has been reported in many recent works on deep model compression that the population risk of a compressed model can be even better than that of the original model. In this paper, an information-theoretic explanation for this population risk improvement phenomenon is provided by jointly studying the decrease in the generalization error and the increase in the empirical risk that results from model compression. It is first shown that model compression reduces an information-theoretic bound on the generalization error, which suggests that model compression can be interpreted as a regularization technique to avoid overfitting. The increase in empirical risk caused by model compression is then characterized using rate distortion theory. These results imply that the overall population risk could be improved by model compression if the decrease in generalization error exceeds the increase in empirical risk. A linear regression example is presented to demonstrate that such a decrease in population risk due to model compression is indeed possible. Our theoretical results further suggest a way to improve a widely used model compression algorithm, i.e., Hessian-weighted *K*-means clustering, by regularizing the distance between the clustering centers. Experiments with neural networks are provided to validate our theoretical assertions.

## 1. Introduction

Although deep neural networks have achieved remarkable success in various domains [1], e.g., computer vision [2], playing games like Go [3], and autonomous driving [4], the improvement of the performance of deep models often comes with deeper layers and more complex network structures, which usually have a large number of parameters. For example, in the application of image classification, it takes over 200 MB to save the parameters of AlexNet [2] and more than 500 MB for VGG-16 net [5]. Hence, it is difficult to port such large models to resource-limited devices such as mobile devices and embedded systems, due to their limited storage, bandwidth, energy, and computational resources.

Due to this reason there has been a flurry of work on compressing deep neural networks (see [6,7,8] for recent surveys). Existing studies mainly focus on designing compression algorithms to reduce the memory and computational cost, while keeping the same level of population risk. In some recent papers [9,10,11,12], aggressive model compression algorithms have been proposed, which require 10% or fewer bits to store the compressed model compared to the storage required by the original model. Surprisingly, it has been observed empirically in these works that the population risk of the compressed model can often be even *better* than that of the original model. This phenomenon is counter-intuitive at first glance, since more compression generally leads to more information loss.

Indeed, a compressed model would usually have a larger empirical risk than the original one, since machine learning methods are usually trained by minimizing the empirical risk. On the other hand, model compression could possibly decrease the generalization error, since it can be interpreted as a regularization technique to avoid overfitting. As the population risk is the sum of the empirical risk and the generalization error, it is possible for the population risk to be reduced by model compression.

### 1.1. Contributions

In this paper, we provide an information-theoretic explanation for the population risk improvement with model compression by jointly characterizing the decrease in generalization error and the increase in empirical risk. Specifically, we focus on the case where the model is compressed based on a pre-trained model.

**Figure 1:**
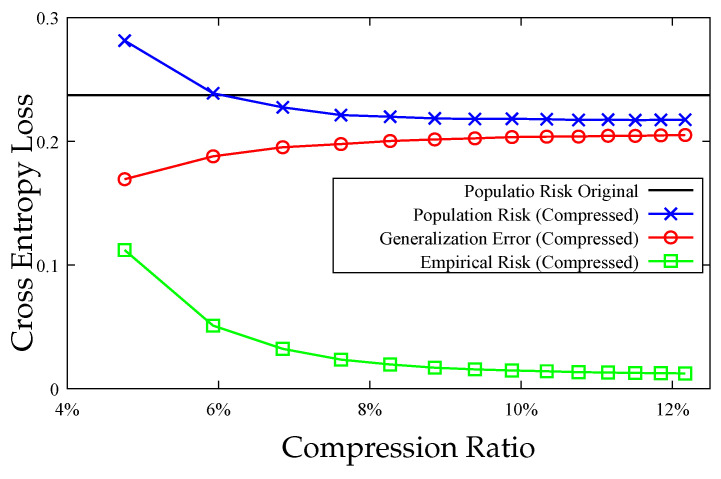
Population risk of the compressed model $\hat{W}$ and the original model *W* vs. compression ratio (ratio of the number of bits used for compressed model to the number of bits used for original model). The generalization error of $\hat{W}$ decreases and the empirical risk of $\hat{W}$ increases with more compression (smaller compression ratio). The population risk of $\hat{W}$ is less than that of *W* for compression ratios larger than 6% in this figure. As the compression ratio goes to 100% (no compression), the population risk of $\hat{W}$ will converge to that of the original model *W*.

We first prove that model compression leads to a tightening of the information-theoretic generalization error bound in [13], and it can therefore be interpreted as a regularization method to reduce overfitting. Furthermore, by defining a distortion metric based on the difference in the empirical risk between the original model obtained by empirical risk minimization (ERM) and compressed models, we use rate distortion theory to characterize the increase in empirical risk as a function of the number of bits *R* used to describe the model. If the decrease in generalization error exceeds the increase in empirical risk, the population risk can be improved. An empirical illustration of this result for the MNIST dataset is provided in Figure 1, where model compression can lead to population risk improvement (details are given in Section 7). To better demonstrate our theoretical results, we investigate the example of linear regression comprehensively, where we develop explicit bounds on the generalization error and the increase in empirical risk.

Our results also suggest a way to improve a method for compression based on Hessian-weighted *K*-means clustering [11] in both scalar and vector case, by regularizing the distance between the clustering centers. Our experiments with neural networks validate our theoretical assertions and demonstrate the effectiveness of the proposed regularizer.

### 1.2. Related Works

There have been many studies on model compression for deep neural networks. The compression could be achieved by varying the training process, e.g., network structure optimization [14], low precision neural networks [15], and  neural networks with binary weights [16,17]. Here we mainly discuss compression approaches that are applied on a pre-trained model.

Pruning, quantization, and matrix factorization are the most popular approaches to compressing pre-trained deep neural networks. The study of pruning algorithms for model compression which remove redundant parameters from neural networks dates back to the 1980s and 1990s [18,19,20]. More recently, an iterative pruning and retraining algorithm to further reduce the size of deep models was proposed in [9,21]. The method of network quantization or weight sharing, i.e., employing a clustering algorithm to group the weights in a neural network, and its variants, including vector quantization [22], soft quantization [23,24], fixed point quantization [25], transform quantization [26], and Hessian weighted quantization [11], have been extensively investigated. Matrix factorization, where low-rank approximation of the weights in neural networks is used instead of the original weight matrix, has also been widely studied in [27,28,29].

All of the aforementioned works demonstrate the effectiveness of their compression methods via comprehensive numerical experiments. Little research has been done to develop a theoretical understanding of how model compression affects performance. In work [30], an information-theoretic view of model compression via rate-distortion theory is provided, with the focus on characterizing the tradeoff between model compression and only the *empirical risk* of the compressed model. In [31,32,33], using a PAC-Bayesian framework, a non-vacuous generalization error bound for compressed model is derived based on its smaller model complexity.

In contrast to these works, instead of focusing on minimizing only the empirical risk as in [30], or minimizing only the generalization error as in [33], we use the mutual information based generalization error bound developed in [13,34] jointly with rate distortion theory to connect analyses of generalization error and empirical risk. This way, we are able to characterize the tradeoff between decrease in generalization error and the increase in empirical risk that results from model compression, and thus provide an understanding as to why model compression can improve the population risk. More importantly, our theoretical studies offer insights on designing practical model compression algorithms.

The rest of the paper is organized as follows. In Section 2, we provide relevant definitions and review relevant results from rate distortion theory. In Section 3, we prove that model compression results in the tightening of an information-theoretic generalization error upper bound. In Section 4, we use rate distortion theory to characterize the tradeoff between the increase in empirical risk and the decrease in generalization error that results from model compression. In Section 5, we quantify this tradeoff for a linear regression model. In Section 6, we discuss how the Hessian-weighted K-means clustering compression approach can be improved by using a regularizer motivated by our theoretical results. In Section 7, we provide some experiments with neural network models to validate our theoretical results and demonstrate the effectiveness of the proposed regularizer.

**Notation** **1.** *For a random variable X generated from a distribution μ, we use $E_{X∼\mu }$ to denote the expectation taken over X with distribution μ. We use $I_{d}$ to denote the d-dimensional identity matrix and ∥A∥ to denote the spectral norm of a matrix A. The cumulant generating function (CGF) of a random variable X is defined as $\Lambda_{X}\left(\lambda \right)≜\ln E\left[e^{\lambda (X-EX)}\right]$. All logarithms are the natural ones.*

## 2. Preliminaries

### 2.1. Review of Rate Distortion Theory

Rate distortion theory, introduced by Shannon [35], is a major branch of information theory that studies the fundamental limits of lossy data compression. It addresses the minimal number of bits per symbol, as measured by the rate *R*, to transmit a random variable *W* such that the receiver can reconstruct *W* without exceeding distortion *D*.

Specifically, let $W^{m}=\left\{W_{1},W_{2},\cdots ,W_{m}\right\}$ denote a sequence of *m* i.i.d. random variables $W_{i}\in W$ generated from a source distribution $P_{W}$. An encoder $f_{m}:W^{m}\to \left\{1,2,\cdots ,M\right\}$ maps the message $W^{m}$ into a codeword, and a decoder $g_{m}:\left\{1,2,\cdots ,M\right\}\to {\hat{W}}^{m}$ reconstructs the message by an estimate ${\hat{W}}^{m}$ from the codeword, where $\hat{W}\subseteq W$ denotes the range of $\hat{W}$. A distortion metric $d:W\times W\to R^{+}$ quantifies the difference between the original and reconstructed messages. The distortion between sequences $w^{m}$ and ${\hat{w}}^{m}$ is defined to be

(1) $$d\left(w^{m},{\hat{w}}^{m}\right)≜\frac{1}{m}\sum_{i=1}^{m}d\left(w_{i},{\hat{w}}_{i}\right).$$

A commonly used distortion metric is the square distortion: $d\left(w,\hat{w}\right)={\left(w-\hat{w}\right)}^{2}$.

**Definition** **1.** *An (m,M,D)-triple is achievable, if there exists a (probabilistic) encoder-decoder pair $\left(f_{m},g_{m}\right)$ such that the alphabet of codeword has size M and the expected distortion $E[d\left(W^{m};g_{m}\left(f_{m}\left(W^{m}\right)\right)\right)]\leq D$.*

Now we define the following rate-distortion and distortion-rate function for lossy data compression.

**Definition** **2.** *The rate-distortion function and the distortion-rate function are defined as*

(2) $$\begin{array}{cc} R(D) & ≜\lim_{m\to \infty }\frac{1}{m}\log_{2}M^{*}\left(m,D\right), \end{array}$$

(3) $$\begin{array}{cc} D(R) & ≜\lim_{m\to \infty }D^{*}\left(m,R\right), \end{array}$$

where $M^{*}\left(m,D\right)≜\min\left\{M:\left(m,M,D\right)\;is\;achievable\right\}$ and $D^{*}\left(m,R\right)≜\min\left\{D:\left(m,2^{mR},D\right)\;is\;achievable\right\}$.

The main theorem of rate distortion theory is as follows.

**Lemma** **1** ([36])**.** *For an i.i.d. source W with distribution $P_{W}$ and distortion function $d(w,\hat{w})$:*

(4) $$\begin{array}{cc} R(D) & =\min_{P_{\hat{W}|W}:E\left[d\left(W,\hat{W}\right)\right]\leq D}I\left(W;\hat{W}\right), \end{array}$$

(5) $$\begin{array}{cc} D(R) & =\min_{P_{\hat{W}|W}:I\left(W;\hat{W}\right)\leq R}E\left[d\left(W,\hat{W}\right)\right], \end{array}$$

*where $I\left(W;\hat{W}\right)≜E_{W,\hat{W}}\left[\ln\frac{P_{W,\hat{W}}}{P_{W}P_{\hat{W}}}\right]$ denotes the mutual information between W and $\hat{W}$.*

The rate-distortion function quantifies the smallest number of bits required to compress the data given the distortion, and the distortion-rate function quantifies the minimal distortion that can be achieved under the rate constraint.

### 2.2. Generalization Error

Consider an instance space Z, a hypothesis space W, and a non-negative loss function $\ell :W\times Z\to R^{+}$. A training dataset $S=\{Z_{1},\cdots ,Z_{n}\}$ consists of *n* i.i.d samples $Z_{i}\in Z$ drawn from an unknown distribution μ. The goal of a supervised learning algorithm is to find an output hypothesis w∈W that minimizes the population risk:

(6) $$L_{\mu }\left(w\right)≜E_{Z∼\mu }\left[\ell \left(w,Z\right)\right].$$

In practice, μ is unknown, and therefore $L_{\mu }\left(w\right)$ cannot be computed directly. Instead, the empirical risk of *w* on the training dataset *S* is studied, which is defined as

(7) $$L_{S}\left(w\right)≜\frac{1}{n}\sum_{i=1}^{n}\ell \left(w,Z_{i}\right).$$

A learning algorithm can be characterized by a randomized mapping from the training dataset *S* to a hypothesis *W* according to a conditional distribution $P_{W|S}$. The (expected) generalization error of a supervised learning algorithm is the expected difference between the population risk of the output hypothesis and its empirical risk on the training dataset:

(8) $$\mathrm{gen}\left(\mu ,P_{W|S}\right)≜E_{W,S}\left[L_{\mu }\left(W\right)-L_{S}\left(W\right)\right],$$

where the expectation is taken over the joint distribution $P_{S,W}=P_{S}⊗P_{W|S}$. The generalization error is used to measure the extent to which the learning algorithm overfits the training data.

## 3. Compression Can Improve Generalization

In this section, we show that lossy compression can lead to a tighter mutual information based generalization error upper bound, which potentially reduces the generalization error of a supervised learning algorithm.

We start from the following lemma which provides an upper bound on the generalization error using the mutual information I(S;W) between training dataset *S* and the output of the learning algorithm *W*.

**Lemma** **2** ([13])**.** *Suppose ℓ(w,Z) is σ-sub-Gaussian (A random variable X is σ-sub-Gaussian if $\Lambda_{X}\left(\lambda \right)\leq \frac{\sigma^{2}\lambda^{2}}{2}$, ∀λ∈R.) under Z∼μ for all w∈W, then*

(9) $$|\mathrm{gen}\left(\mu ,P_{W|S}\right)|\leq \sqrt{\frac{2\sigma^{2}}{n}I\left(S;W\right)}.$$

Compression can be viewed as a post-processing of the output of a learning algorithm. The output model *W* generated by a learning algorithm can be quantized, pruned, factorized, or even perturbed by noise, which results in a compressed model $\hat{W}$. Assume that the compression algorithm is only based on *W* and can be described by a conditional distribution $P_{\hat{W}|W}$. Then the following Markov chain holds: $S\to W\to \hat{W}$. By the data processing inequality,

$$I\left(S;\hat{W}\right)\leq \min\left\{I\left(W;\hat{W}\right),I\left(S,W\right)\right\}.$$

Thus, we have the following theorem characterizing the generalization error of the compressed model.

**Theorem** **1.** *Consider a learning algorithm $P_{W|S}$, a compression algorithm $P_{\hat{W}|W}$, and suppose $\ell (\hat{w},Z)$ is σ-sub-Gaussian under Z∼μ for all $\hat{w}\in \hat{W}$. Then*

(10) $$|\mathrm{gen}\left(\mu ,P_{\hat{W}|S}\right)|\leq \sqrt{\frac{2\sigma^{2}}{n}\min\left\{I\left(W;\hat{W}\right),I\left(S,W\right)\right\}}.$$

Note that the generalization error upper bound in Theorem 1 for the compressed model is always no greater than the one in Lemma 2. This allows for the interpretation of compression as a regularization technique to reduce the generalization error.

## 4. Generalization Error and Model Distortion

In this section, we define a distortion metric in model compression that allows us to relate the distortion (the increase in empirical risk) due to compression with the reduction in the generalization error bound discussed in Section 3.

### 4.1. Distortion Metric in Model Compression

The expected population risk of a model *W* can be written as

(11) $$E_{W}\left[L_{\mu }\left(W\right)\right]=E\left[L_{S}\left(W\right)\right]+\mathrm{gen}\left(\mu ,P_{W|S}\right),$$

where the first term, which is the expected empirical risk, reflects how well the model *W* fits the training data, while the second term demonstrates how well the model generalizes. In the empirical risk minimization framework, we control both terms by (1) minimizing the empirical risk of *W* directly or using other stochastic optimization algorithms, and (2) using regularization methods to control the generalization error, e.g., early stopping and dropout [1].

Now, consider the expected population risk of the compressed model $\hat{W}$:

(12) $$\begin{array}{cc} E_{\hat{W}}\left[L_{\mu }\left(\hat{W}\right)\right]\; & =E[L_{\mu }\left(\hat{W}\right)-L_{S}\left(\hat{W}\right)+L_{S}\left(\hat{W}\right)-L_{S}\left(W\right)+L_{S}\left(W\right)]\\ \; & =E\left[L_{S}\left(W\right)\right]+\mathrm{gen}\left(\mu ,P_{\hat{W}|S}\right)+E\left[L_{S}\left(\hat{W}\right)-L_{S}\left(W\right)\right]. \end{array}$$

Compared with (11), we note that the first empirical risk term is independent of the compression algorithm, the second generalization error term can be upper bounded by Theorem 1, and the third term $E[L_{S}\left(\hat{W}\right)-L_{S}\left(W\right)]$ quantifies the increase in the empirical risk if we use the compressed model $\hat{W}$ instead of the original model *W*. We then define the following distortion metric for model compression:

(13) $$d_{S}\left(w,\hat{w}\right)≜L_{S}\left(\hat{w}\right)-L_{S}\left(w\right),$$

which is the difference in the empirical risk between the compressed model $\hat{W}$ and the original model *W*. In general, function $d_{S}\left(w,\hat{w}\right)$ is not always non-negative. However, for ERM solution *W*, which is obtained by minimizing the empirical risk $L_{S}\left(W\right)$, $d_{S}\left(w,\hat{w}\right)\geq 0$, which ensures that $d_{S}\left(w,\hat{w}\right)$ is a valid distortion metric. By Theorem 1, it follows that

(14) $$\begin{array}{c} E_{S,W,\hat{W}}\left[L_{\mu }\left(\hat{W}\right)-L_{S}\left(W\right)\right]\leq \sqrt{\frac{2\sigma^{2}}{n}I\left(W;\hat{W}\right)}+E_{S,W,\hat{W}}\left[d_{S}\left(\hat{W},W\right)\right]≜L_{S,W}\left(P_{\hat{W}|W}\right), \end{array}$$

where $L_{S,W}\left(P_{\hat{W}|W}\right)$ is an upper bound on the expected difference between the population risk of $\hat{W}$ and the empirical risk of the original model *W* on training dataset *S*. Note that $L_{S}\left(W\right)$ is independent of the compression algorithm. Therefore, the bound in (14) can be viewed as an upper bound of the population risk of the compressed model $\hat{W}$.

### 4.2. Population Risk Improvement

By Lemma 1, the smallest distortion that can be achieved at rate *R* is $D\left(R\right)=\min_{I(W;\hat{W})\leq R}E_{S,W,\hat{W}}\left[d_{S}\left(\hat{W},W\right)\right]$. Thus, the tightest bound in (14) that can be achieved at rate *R* is given in the following theorem.

**Theorem** **2.** *Suppose the assumptions in Theorem 1 hold, $P_{W|S}$ minimizes the empirical risk $L_{S}\left(W\right)$, and $I(W;\hat{W})=R$, then*

(15) $$\begin{array}{c} \min_{P_{\hat{W}|W}:I\left(W;\hat{W}\right)=R}E_{S,W,\hat{W}}\left[L_{\mu }\left(\hat{W}\right)-L_{S}\left(W\right)\right]\leq \sqrt{\frac{2\sigma^{2}}{n}R}+D\left(R\right). \end{array}$$

From the properties of the distortion-rate function [36], we know that D(R) is a decreasing function of *R*. Thus, we see that as *R* decreases the first term in (15), which corresponds to the generalization error, decreases, while the second term, which corresponds to the empirical risk, increases. Due to this tradeoff, it may be possible for the bound in (15) to be smaller due to compression, i.e., using a smaller rate *R*. This indicates that the population risk could improve with compression algorithm, which minimizes the upper bound $L_{S,W}\left(P_{\hat{W}|W}\right)$.

**Remark** **1.** *In order to conclude definitively that the population risk can be improved with compression, we need to find a lower bound (as a function of R) to match (at least in the order sense) the upper bound in Theorem 2. This appears to be difficult to construct in general. One approach might be to use the same decomposition as in (12) and develop lower bounds for $\min_{I(W;\hat{W})=R}\mathrm{gen}\left(\mu ,P_{\hat{W}|S}\right)$ and $\min_{I(W;\hat{W})=R}E_{S,W,\hat{W}}\left[d_{S}\left(\hat{W},W\right)\right]$ independently. However, such an approach runs into the following issues: (1) such a lower bound would be loose since the compression algorithm $P_{\hat{W}|W}$ that minimizes generalization error, the one that minimizes the distortion, and the one that minimizes the sum of the two can be quite different; and (2) a lower bound for generalization error needs to be developed, which appears to be difficult, with existing literature mainly focusing on lower bounding the excess risk, e.g., [37].*

As will be shown in Section 7, we can actually improve the population risk with a well designed compression algorithm in practical applications.

## 5. Example: Linear Regression

In this section, we comprehensively explore the example of linear regression to get a better understanding of the results in Section 4. To this end, we develop explicit upper bounds for generalization error and distortion-rate function D(R). All the proofs of the lemmas and theorems are provided in the Appendix A, Appendix B, Appendix C and Appendix D.

Suppose that the dataset $S=\left\{Z_{1},\cdots ,Z_{n}\right\}=\left\{\left(X_{1},Y_{1}\right),\cdots ,\left(X_{n},Y_{n}\right)\right\}$ is generated from the following linear model with weight vector $w^{*}=\left(w^{*(1)},\cdots ,w^{*(d)}\right)\in R^{d}$,

(16) $$Y_{i}=X_{i}^{⊤}w^{*}+\varepsilon_{i},\;i=1,\cdots ,n,$$

where $X_{i}$’s are i.i.d. *d*-dimensional random vectors with distribution $N(0,\Sigma_{X})$, and $\varepsilon_{i}∼N\left(0,{\sigma^{\prime }}^{2}\right)$ denotes i.i.d. Gaussian noise. We adopt the mean squared error as the loss function, and the corresponding empirical risk on *S* is

(17) $$L_{S}\left(w\right)=\frac{1}{n}\sum_{i=1}^{n}{\left(Y_{i}-X_{i}^{⊤}w\right)}^{2}=\frac{1}{n}{∥Y-X^{⊤}w∥}_{2}^{2},$$

for $w\in W=R^{d}$, where $X\in R^{d\times n}$ denotes all the input samples, and $Y\in R^{n}$ denotes the responses. If n>d, the ERM solution is

(18) $$W={\left(XX^{⊤}\right)}^{-1}XY,$$

which is deterministic given *S*. Its generalization error can be computed exactly as in the following lemma (see Appendix A for detailed proof).

**Lemma** **3.** *If n>d+1, then*

(19) $$\mathrm{gen}\left(\mu ,P_{W|S}\right)=\frac{{\sigma^{\prime }}^{2}d}{n}\left(2+\frac{d+1}{n-d-1}\right).$$

### 5.1. Information-Theoretic Generalization Bounds for Compressed Linear Model

We note that the mutual information based bound in Lemma 2 is not applicable for this linear regression model, since *W* is a deterministic function of *S*, and I(S;W)=∞. However, this issue can be resolved if we post-process the ERM solution *W* by a compression algorithm and upper bound the generalization error by $I(\hat{W};W)$ as shown in Theorem 1.

Consider a compression algorithm, which maps the original weights $W\in R^{d}$ to the compressed model $\hat{W}\in \hat{W}\subseteq R^{d}$. For a fixed and compact $\hat{W}$, we define

(20) $$C\left(w^{*}\right)≜\underset{\hat{w}\in \hat{W}}{\sup}{∥\hat{w}-w^{*}∥}_{2}^{2},$$

which measures the largest distance between the reconstruction $\hat{w}$ and the optimal weights $w^{*}$. The following proposition provides an upper bound on the generalization error of the compressed model $\hat{W}$, and the detailed proof is provided in Appendix B.

**Proposition** **1.** *Consider the ERM solution $W={\left(XX^{⊤}\right)}^{-1}XY$, and suppose $\hat{W}$ is compact, then*

(21) $$\mathrm{gen}\left(\mu ,P_{\hat{W}|S}\right)\leq 2\sigma_{\ell }^{*2}\sqrt{\frac{I(W;\hat{W})}{n}},$$

where $\sigma_{\ell }^{*2}≜C\left(w^{*}\right)∥\Sigma_{X}∥+{\sigma^{\prime }}^{2}$.

### 5.2. Distortion-Rate Function for Linear Model

We now provide an upper bound on the distortion-rate function D(R) for the linear regression model. Note that $\nabla L_{S}\left(W\right)=0$, since *W* minimizes the empirical risk. The Hessian matrix of the loss function is

(22) $$H_{S}\left(W\right)=\frac{1}{n}XX^{⊤},$$

which is not a function of *W*. Then, the distortion function can be written as:

(23) $$\begin{array}{cc} E_{S,W,\hat{W}}\left[d_{S}\left(\hat{W},W\right)\right]\; & =E_{S,W,\hat{W}}\left[L_{S}\left(\hat{W}\right)-L_{S}\left(W\right)\right]\\ \; & =E_{S,W,\hat{W}}\left[{\left(\hat{W}-W\right)}^{⊤}\frac{1}{n}XX^{⊤}\left(\hat{W}-W\right)\right]. \end{array}$$

The following theorem characterizes upper bounds for R(D) and D(R) for linear regression.

**Proposition** **2.** *For the ERM solution $W={\left(XX^{⊤}\right)}^{-1}XY$, we have*

(24) $$\begin{array}{cc} R(D) & \leq \frac{d}{2}{\left(\ln\frac{d{\sigma^{\prime }}^{2}}{(n-d-1)D}\right)}^{+},\;D\geq 0, \end{array}$$

(25) $$\begin{array}{cc} D(R) & \leq \frac{d{\sigma^{\prime }}^{2}}{n-d-1}e^{-\frac{2R}{d}},\;R\geq 0, \end{array}$$

*where ${\left(x\right)}^{+}=\max\left\{0,x\right\}$.*

**Proof sketch**. The proof of the upper bound for R(D) is based on considering a Gaussian random vector which has the same mean and covariance matrix as *W*. In addition, the upper bound is achieved when $W-\hat{W}$ is independent of the dataset *S* with the following conditional distribution,

(26) $$P_{\hat{W}|W}=N\left(\left(1-\alpha \right)W+\alpha w^{*},\left(1-\alpha \right)\frac{D}{d}\Sigma_{X}^{-1}\right),$$

where $\alpha ≜\frac{nD}{d{\sigma^{\prime }}^{2}}\leq 1$. Note that this “compression algorithm” requires the knowledge of optimal weights $w^{*}$, which is unknown in practice.

The details can be found in Appendix C.    □

**Remark** **2.** *As shown in [38], if $n>d/\epsilon^{2}$, $∥\frac{1}{n}XX^{⊤}-\Sigma_{X}∥\leq \epsilon$ holds with high probability. Then, the following lower bound on R(D) holds if we can approximate $\frac{1}{n}XX^{⊤}$ in (23) using $\Sigma_{X}$,*

(27) $$R\left(D\right)≳\frac{d}{2}{\left(\ln\frac{d{\sigma^{\prime }}^{2}}{(n-d-1)D}\right)}^{+}-D\left(P_{W}∥P_{W_{G}}\right),$$

*where $W_{G}$ denotes a Gaussian random vector with the same mean and variance as W. The details can be found in Appendix D.*

Combing Propositions 1 and 2, we have the following result.

**Corollary** **1.** *Under the same assumptions as in Proposition 1, we have*

(28) $$\begin{array}{c} \min_{P_{\hat{W}|W}:I\left(W;\hat{W}\right)=R}E_{S,W,\hat{W}}\left[L_{\mu }\left(\hat{W}\right)-L_{S}\left(W\right)\right]\leq 2\sigma_{\ell }^{*2}\sqrt{\frac{R}{n}}+\frac{d{\sigma^{\prime }}^{2}}{n-d-1}e^{-\frac{2R}{d}},\;R\geq 0. \end{array}$$

In (28) the first term corresponds to the generalization error, which decreases with compression, and the second term corresponds to the empirical risk, which increases with compression.

### 5.3. Evaluation and Visualization

In the following plots, we generate the training dataset *S* using the linear model in (16) by letting d=50, n=80, $\Sigma_{X}=I_{d}$ and ${\sigma^{\prime }}^{2}=1$. We consider the following two compression algorithms. The first one is the conditional distribution $P_{\hat{W}|W}$ in the proof of achievability (26), which requires the knowledge of $w^{*}$ and is denoted as “Oracle”. The second one is the well-known *K*-means clustering algorithm, where the weights in *W* are grouped into *K* clusters and represented by the cluster centers in the reconstruction $\hat{W}$. By changing the number of clusters *K*, we can control the rate *R*, i.e., $I(W;\hat{W})$. We average the performance and estimate $I(W;\hat{W})$ of these algorithms with 10,000 Monte-Carlo trials in the simulation.

We note that $I(W;\hat{W})$ is equal to the number of bits used in compression only in the asymptotic regime of large number of samples. In practice, we may have only one sample of the weights *W*, and therefore $I(W;\hat{W})$ simply measures the extent to which compression is performed by the compression algorithm.

**Figure 2:**
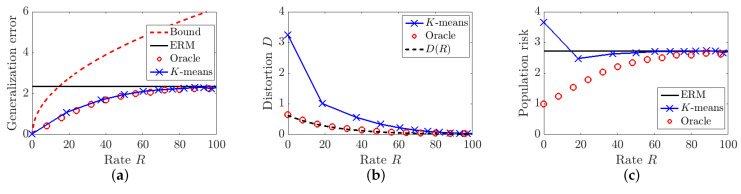
Comparison of three different quantities for linear regression as a function of rate *R* in bits. (**a**) Generalization error. (**b**) Distortion. (**c**) Population risk.

In Figure 2a, we plot the generalization error bound in Proposition 1 as a function of the rate *R* and compare the generalization errors of the Oracle and *K*-means algorithms. It can be seen that Proposition 1 provides a valid upper bound for the generalization error, but this bound is tight only when *R* is small. Moreover, both compression algorithms can achieve smaller generalization errors compared to that of the ERM solution *W*, which validates the result in Theorem 1.

Figure 2b plots the upper bound on the distortion-rate function in Theorem 2 and the distortions achieved by the Oracle and *K*-means algorithms. The distortion of the Oracle decreases as we increase the rate *R* and matches the D(R) function well. However, there is a large gap between the distortion achieved by *K*-means algorithms and D(R). One possible explanation is that since $w^{*}$ is unknown, it is impossible for the *K*-means algorithm to learn the optimal cluster center with only one sample of *W*. Even if we view $W^{\left(j\right)},j=1,\cdots ,d$ as i.i.d. samples from the same distribution, there is still a gap between the distortion achieved by the *K*-means algorithm and the optimal quantization as studied in [39].

We plot the population risks of the ERM solution *W*, the Oracle, and *K*-means algorithms in Figure 2c. It is not surprising that the Oracle algorithm achieves a small population risk, since $\hat{W}$ is a function of $w^{*}$ and $\hat{W}=w^{*}$ when R=0. However, it can be seen that the *K*-means algorithm achieves a smaller population risk than the original model *W*, since the decrease in generalization error exceeds the increase in empirical risk, when we use fewer clusters in the *K*-means algorithm, i.e., a smaller rate *R*. We note that the minimal population risk is achieved when K=2, since we initialize $w^{*}$ so that $w^{*(i)}$, 1≤i≤d, can be well approximated by two cluster centers.

## 6. Clustering Algorithm Minimizing $L_{S,W}$

In this section, we propose an improvement of the Hessian-weighted (HW) *K*-means clustering algorithm [11] for model compression by regularizing the distance between the cluster centers, which minimizes the upper bound $L_{S,W}\left(P_{\hat{W}|W}\right)$, as suggested by our theoretical results in Section 4.

### 6.1. Hessian-Weighted K-Means Clustering

The goal of HW *K*-means is to minimize the distortion on the empirical risk $d_{S}\left(\hat{W},W\right)$, which has the following Taylor series approximation:

(29) $$\begin{array}{cc} \; & d_{S}\left(\hat{W},W\right)\approx {\left(\hat{W}-W\right)}^{T}\nabla L_{S}\left(W\right)+\frac{1}{2}{\left(\hat{W}-W\right)}^{T}H_{S}\left(W\right)\left(\hat{W}-W\right), \end{array}$$

where $H_{S}\left(W\right)$ is the Hessian matrix. Assuming that *W* is a local minimum of $L_{S}\left(W\right)$ (ERM solution) and $\nabla L_{S}\left(W\right)\approx 0$, the first term can be ignored. Furthermore, the Hessian matrix $H_{S}\left(W\right)$ can be approximated by a diagonal matrix, which further simplifies the objective to $d_{S}\left(\hat{W},W\right)\approx \sum_{j=1}^{d}h^{\left(j\right)}{\left(W^{\left(j\right)}-{\hat{W}}^{\left(j\right)}\right)}^{2}$, where $h^{\left(j\right)}$ is the *j*-th diagonal element of the Hessian matrix.

Given network parameters $w=\{w^{\left(1\right)},\cdots ,w^{\left(d\right)}\}$, the HW *K*-means clustering algorithm [11] partitions them into *K* disjoint clusters, using a set of cluster centers $c=\{c^{\left(1\right)},\cdots ,c^{\left(K\right)}\}$, and a cluster assignment $C=\left\{C^{\left(1\right)},\cdots ,C^{\left(K\right)}\right\}$, while solving the following optimization problem:

(30) $$\min\sum_{k=1}^{K}\sum_{w^{\left(j\right)}\in C^{\left(k\right)}}h^{\left(j\right)}{\left|w^{\left(j\right)}-c^{\left(k\right)}\right|}^{2}.$$

### 6.2. Diameter Regularization

In contrast to HW *K*-means which only cares about empirical risk, our goal is to obtain as small a population risk as possible by minimizing the upper bound

(31) $$L_{S,W}\left(P_{\hat{W}|W}\right)=\sqrt{\frac{2\sigma^{2}}{n}I\left(W;\hat{W}\right)}+E\left[d_{S}\left(\hat{W},W\right)\right].$$

Here, we let the number of clusters *K* to be an input argument of the algorithm, so that $I\left(W;\hat{W}\right)\leq \log_{2}K$, and we want to minimize $L_{S,W}\left(P_{\hat{W}|W}\right)$ by carefully designing the reconstructed weights given *K*, i.e., by choosing cluster centers $\left\{c^{\left(1\right)},\cdots ,c^{\left(K\right)}\right\}$. Then, minimizing the sub-Gaussian parameter σ is one way to control the generalization error of the compression algorithm. Recall that in Proposition 1, we have

(32) $$\mathrm{gen}\left(\mu ,P_{\hat{W}|S}\right)\leq 2\left(C\left(w^{*}\right)∥\Sigma_{X}∥+{\sigma^{\prime }}^{2}\right)\sqrt{\frac{I(W;\hat{W})}{n}},$$

where the sub-Gaussian parameter is related to $C\left(w^{*}\right)=\sup_{\hat{w}\in \hat{W}}{∥\hat{w}-w^{*}∥}_{2}^{2}$ in linear regression. Note that this quantity can be interpreted as the diameter of the set W. Since the ground truth $w^{*}$ is unknown in practice, we then propose the following diameter regularization by approximating $C(w^{*})$ in (32) by

(33) $$\beta \max_{k_{1},k_{2}}{\left|c^{\left(k_{1}\right)}-c^{\left(k_{2}\right)}\right|}^{2},\;\beta \geq 0,$$

where β is a parameter controls the penalty term and can be selected by cross validation in practice. Our diameter-regularized Hessian-weighted (DRHW) *K*-means algorithm solves the following optimization problem:

(34) $$\min\sum_{k=1}^{K}\sum_{w^{\left(j\right)}\in C^{\left(k\right)}}h^{\left(j\right)}|w^{\left(j\right)}-c^{\left(k\right)}{|}^{2}+\beta \max_{k_{1},k_{2}}{\left|c^{\left(k_{1}\right)}-c^{\left(k_{2}\right)}\right|}^{2}.$$

Such an optimization problem can be easily extended to the vector case which leads to a vector quantization algorithm. Suppose that we group the *d*-dimensional weights $w=\{w^{\left(1\right)},\cdots ,w^{\left(d\right)}\}$ into $d^{\prime }=d/m$ vectors with length *m*, i.e., $\left\{w^{\left(1\right)},\cdots ,w^{\left(d^{\prime }\right)}\right\}$, $w^{\left(j\right)}\in R^{m}$, then our goal is to find cluster centers $c^{k}\in R^{m}$ and assignments minimizing the following cost function:

(35) $$\min\sum_{k=1}^{K}\sum_{w^{\left(j\right)}\in C^{\left(k\right)}}{\left(w^{\left(j\right)}-c^{\left(k\right)}\right)}^{⊤}H^{\left(j\right)}\left(w^{\left(j\right)}-c^{\left(k\right)}\right)+\beta \max_{k_{1},k_{2}}{∥c^{\left(k_{1}\right)}-c^{\left(k_{2}\right)}∥}_{2}^{2},$$

where $H^{\left(j\right)}$ is the diagonal Hessian matrix corresponding to the vector $w^{\left(j\right)}$. An iterative algorithm to solve the above optimization problem for vector quantization is provided in Algorithm 1.

The algorithm alternates between minimizing the objective function over the cluster centers and the assignments. In the Assignment step, we first fix centers and assign each $w^{\left(j\right)}$ to its nearest neighbor. We then fix assignments and update the centers by the weighted mean of each cluster in the Update step. For the farthest pair of centers, the diameter regularizer pushes them toward each other, so that the output centers have potentially smaller diameters than those of regular *K*-means. We note that the time complexity of the proposed diameter-regularized Hessian weighted *K*-means algorithm is the same as that of the original *K*-means algorithm.
**Algorithm 1** Diameter-regularized Hessian weighted *K*-means in vector case**Input:** Weights vector $\left\{w^{\left(1\right)},\cdots ,w^{\left(d^{\prime }\right)}\right\}$, Hessian matrices $\left\{H^{\left(1\right)},\cdots ,H^{\left(d^{\prime }\right)}\right\}$, diameter regularizer β>0, number of clusters *K*, iterations *T* **Initialize** the *K* cluster centers $\left\{c_{0}^{\left(1\right)},\cdots ,c_{0}^{\left(K\right)}\right\}$ randomly **for** t=1 to *T* **do**  **Assignment step:**  Initialize $C_{t}^{\left(k\right)}=\emptyset$ for all k∈[K].  **for** j=1 to $d^{\prime }$ **do**   Assign $w^{\left(j\right)}$ to the nearest cluster center, i.e., find $k_{t}^{\left(j\right)}=\arg\min_{k\in [K]}{∥w^{\left(j\right)}-c_{t-1}^{\left(k\right)}∥}_{2}^{2}$ and let
(36)$$\begin{array}{c} C_{t}^{\left(k_{t}^{\left(j\right)}\right)}\leftarrow C_{t}^{\left(k_{t}^{\left(j\right)}\right)}\cup \left\{w^{\left(j\right)}\right\} \end{array}$$  **end for**  **Update step:**  Find current farthest pair of centers $\left(k_{1},k_{2}\right)=\arg\max_{k_{1},k_{2}}{∥c_{t-1}^{\left(k_{1}\right)}-c_{t-1}^{\left(k_{2}\right)}∥}_{2}^{2}$.  Update $c_{t}^{\left(k_{1}\right)}$ and $c_{t}^{\left(k_{2}\right)}$ by
(37)$$\begin{array}{c} c_{t}^{\left(k_{1}\right)}={\left(\sum_{w^{\left(j\right)}\in C_{t}^{\left(k_{1}\right)}}H^{\left(j\right)}+\beta I_{m}\right)}^{-1}\left(\sum_{w^{\left(j\right)}\in C_{t}^{\left(k_{1}\right)}}H^{\left(j\right)}w^{\left(j\right)}+\beta c_{t}^{\left(k_{2}\right)}\right)\\ c_{t}^{\left(k_{2}\right)}={\left(\sum_{w^{\left(j\right)}\in C_{t}^{\left(k_{2}\right)}}H^{\left(j\right)}+\beta I_{m}\right)}^{-1}\left(\sum_{w^{\left(j\right)}\in C_{t}^{\left(k_{2}\right)}}H^{\left(j\right)}w^{\left(j\right)}+\beta c_{t}^{\left(k_{1}\right)}\right) \end{array}$$  **for** k=1 to *K*, $k\notin \{k_{1},k_{2}\}$ **do**  Update the cluster centers by
(38)$$\begin{array}{c} c_{t}^{\left(k\right)}={\left(\sum_{w^{\left(j\right)}\in C_{t}^{\left(k\right)}}H^{\left(j\right)}\right)}^{-1}\left(\sum_{w^{\left(j\right)}\in C_{t}^{\left(k\right)}}H^{\left(j\right)}w^{\left(j\right)}\right) \end{array}$$  **end for** **end for****Output:** centers $\left\{c_{T}^{\left(1\right)},\cdots ,c_{T}^{\left(K\right)}\right\}$ and assignments $\left\{C_{T}^{\left(1\right)},\cdots ,C_{T}^{\left(K\right)}\right\}$.

## 7. Experiments

In this section, we provide some real-world experiments to validate our theoretical assertions and the DRHW *K*-means algorithm. (The code for our experiments is available at the following link https://github.com/wgao9/weight-quant (accessed on 13 August 2021)) Our experiments include compression of: (i) a three-layer fully connected network on the MNIST dataset [40]; and (ii) a convolutional neural network with five convolutional layers and three linear layers on the CIFAR10 dataset [41] (We downloaded the pre-trained model in PyTorch from https://github.com/aaron-xichen/pytorch-playground (accessed on 13 August 2021)).

In Theorem 1, an upper bound on the *expected* generalization error is provided, and therefore we independently train 50 different models (with the same structure but different parameter initializations) using different subset of training samples, and average the results. We use 10% of the training data to train the model for MNIST and use 20% of the training data to train the model for CIFAR10. For each experiment, we use the same number of clusters for each convolutional layer and fully connected layer.

In the following experiments, we plot the cross entropy loss as a function of compression ratio. Note that compression ratio can be controlled by changing the number of clusters *K* in the quantization algorithm. To see this, suppose that the neural networks have total of *d* parameters that need to be compressed, and each parameter is of *b* bits. Let $C^{\left(k\right)}$ be the set of weights in cluster *k* and let $b_{k}$ be the number of bits of the codeword assigned to the network parameters in cluster *k* for 1≤k≤K. For a lookup table to decode quantized values, we need Kb bits to store all the reconstructed weights, i.e., cluster centers $c=\{c^{\left(1\right)},\cdots ,c^{\left(K\right)}\}$. Then, the compression ratio is given by

(39) $$\mathrm{Compression}\;\mathrm{Ratio}=\frac{\sum_{k=1}^{K}\left|C^{\left(k\right)}\right|b_{k}+Kb}{db},$$

where |·| denotes the number of elements in the set. In our experiments, we use a variable-length code such as the Huffman code to compute the compression ratio under different numbers of clusters *K*.

**Figure 3:**
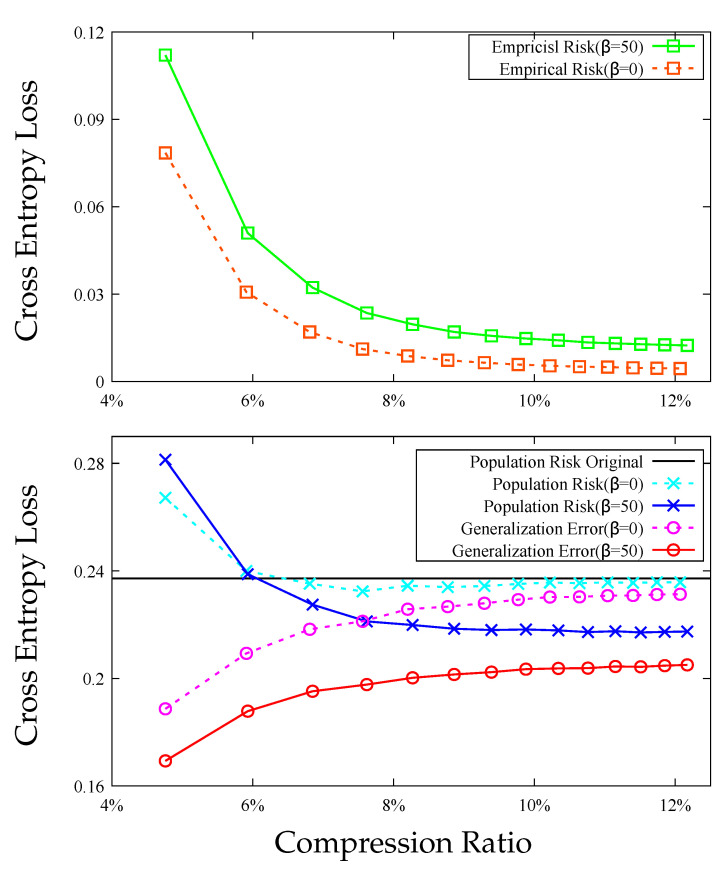
Comparison between DRHW *K*-means (β=50) and HW *K*-means (β=0) on MNIST. **Top**: empirical risks. **Bottom**: population risks and generalization errors.

**Figure 4:**
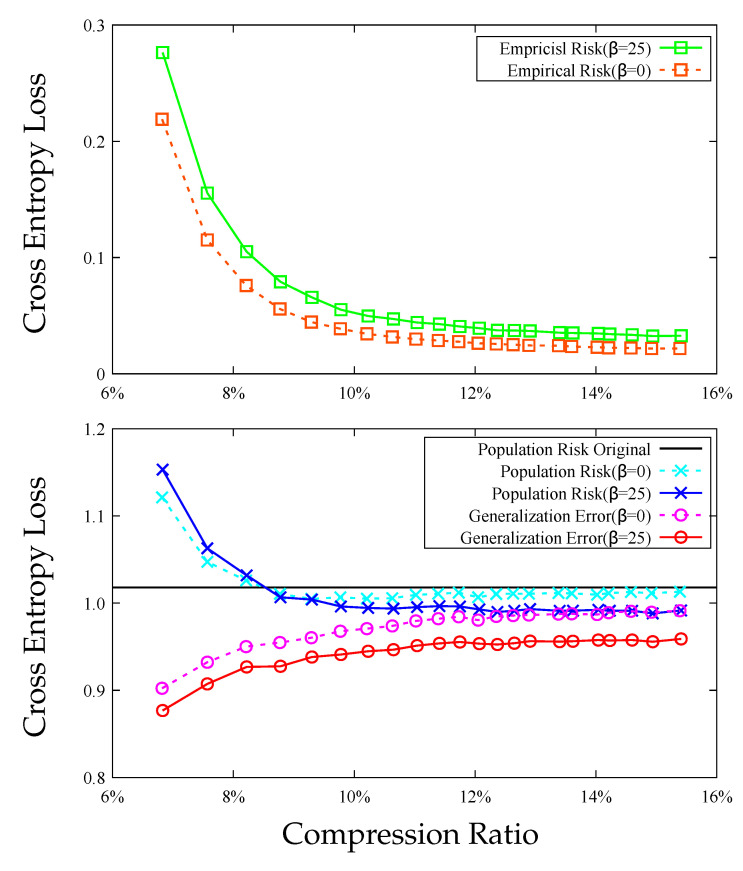
Comparison between DRHW *K*-means (β=25) and HW *K*-means (β=0) on CIFAR10. **Top**: empirical risks. **Bottom**: population risks and generalization errors.

In Figure 3 and Figure 4, we compare the scalar DRHW *K*-means algorithm with the scalar HW *K*-means algorithm for different compression ratios on the MNIST and CIFAR10 datasets. Both figures demonstrate that the compression algorithm increases the empirical risk but decreases the generalization error, and the net effect is that the both compressed models have smaller population risks than those of the original models. More importantly, the DRHW *K*-means algorithm produces a compressed model that has a better population risk than that of the HW *K*-means algorithm.

**Figure 5:**
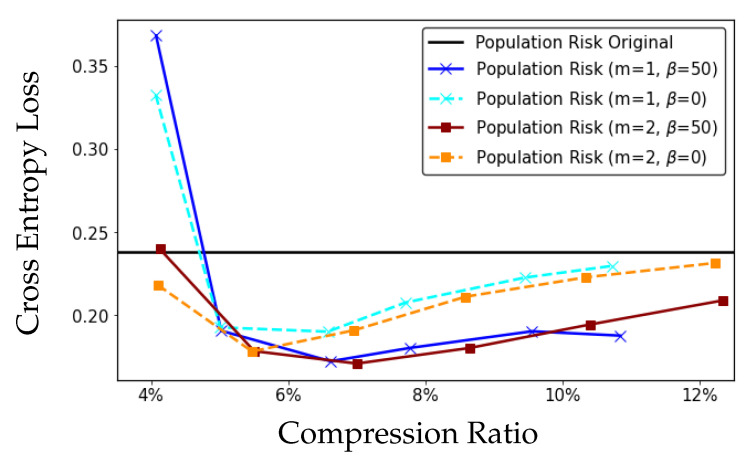
Comparison between scalar DRHW *K*-means (m=1) and vector DRHW *K*-means (m=2) on the MNIST dataset.

In Figure 5, we compare the population risk of scalar DRHW *K*-means algorithm and that of the vector DRHW *K*-means algorithm with block length m=2 for different compression ratios on the MNIST dataset. It can be seen from the figure that the improvement by using vector quantization (m=2) is quite modest, which implies that the dependence between the weights $W^{\left(j\right)}$ is weak. However, we can still observe the improvement of adding the diameter regularizer in vector DRHW *K*-means algorithm by comparing the curves with β=50 and β=0.

**Figure 6:**
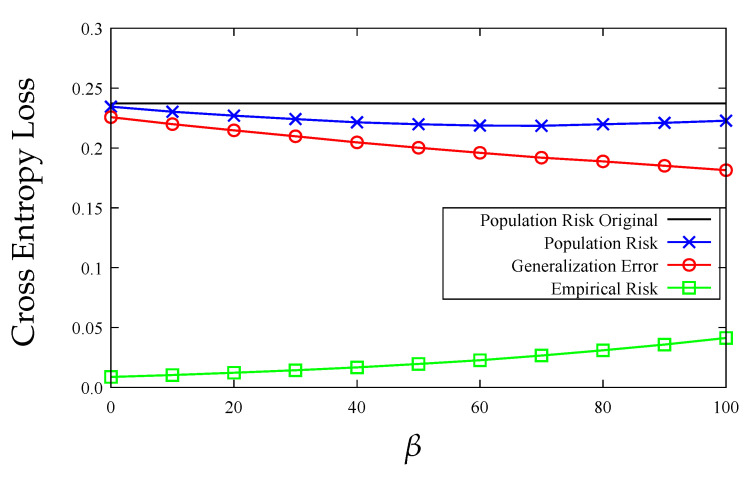
DRHW *K*-means with different β on the MNIST dataset with K=7.

In Figure 6, we demonstrate how β affects the performance of our diameter-regularized Hessian-weighted *K*-means algorithm in scalar case. It can be seen that as β increases, the generalization error decreases and the distortion in empirical risk increases, which validates the idea that this proposed diameter regularizer can be used to reduce the generalization error. The value of β that results in the best population risk therefore can be chosen via cross-validation in practice.

## 8. Conclusions

In this paper, we have provided an information-theoretical understanding of how model compression affects the population risk of a compressed model. In particular, our results indicate that model compression may increase the empirical risk but decrease the generalization error. Therefore, it might be possible to achieve a smaller population risk via model compression. Our experiments validate these theoretical findings. Furthermore, we showed how our information-theoretic bound on the population risk can be used to optimize practical compression algorithms.

We note that our results could be applied to improve other compression algorithms, such as pruning and matrix factorization. Moreover, we believe that the information-theoretic analysis adopted here could be generalized to characterize a similar tradeoff between the generalization error and empirical risk in other applications beyond compressing pre-trained models, e.g., distributed optimization [42] and low precision training [15].

## Data Availability

Data and code can be found in Section 7.

## Appendix A. Proof of Lemma 3

Let $\tilde{Z}=\left(\tilde{X},\tilde{Y}\right)$, $\tilde{X}\in R^{d}$ and $\tilde{Y}\in R$ denote an independent copy of the training sample $Z_{i}$. Then, it can be shown that

(A1) $$\begin{array}{cc} \mathrm{gen}(\mu ,P_{W|S})\; & =E_{W,S}\left[L_{\mu }\left(W\right)-L_{S}\left(W\right)\right]\\ \; & =E_{W,S}\left[E_{\tilde{Z}}\left[{\left(\tilde{Y}-{\tilde{X}}^{⊤}W\right)}^{2}\right]-\frac{1}{n}{∥Y-X^{⊤}W∥}_{2}^{2}\right]\\ \; & =E_{S}\left[E_{\tilde{Z}}\left[{\left(\tilde{Y}-{\tilde{X}}^{⊤}{\left(XX^{⊤}\right)}^{-1}XY\right)}^{2}\right]-\frac{1}{n}{∥Y-X^{⊤}{\left(XX^{⊤}\right)}^{-1}XY∥}_{2}^{2}\right], \end{array}$$

where $\tilde{Y}={\tilde{X}}^{⊤}w^{*}+\tilde{\varepsilon }$ and $Y=X^{⊤}w^{*}+\varepsilon$. Then, we have

(A2) $$\begin{array}{cc} \mathrm{gen}(\mu ,P_{W|S}) & =E_{\varepsilon ,\tilde{\varepsilon },X,\tilde{X}}\left[{\left(\tilde{\varepsilon }-{\tilde{X}}^{⊤}{\left(XX^{⊤}\right)}^{-1}X\varepsilon \right)}^{2}\right]-\frac{1}{n}E_{\varepsilon ,X}\left[∥\varepsilon -X^{⊤}{\left(XX^{⊤}\right)}^{-1}{X\varepsilon ∥}_{2}^{2}\right]\\ \; & =E_{\varepsilon ,X,\tilde{X}}\left[\varepsilon^{⊤}X^{⊤}{\left(XX^{⊤}\right)}^{-1}\tilde{X}{\tilde{X}}^{⊤}{\left(XX^{⊤}\right)}^{-1}X\varepsilon +\frac{1}{n}\varepsilon^{⊤}X^{⊤}{\left(XX^{⊤}\right)}^{-1}X\varepsilon \right]\\ \; & =E_{\varepsilon ,X}\left[\mathrm{Tr}(X^{⊤}{\left(XX^{⊤}\right)}^{-1}\Sigma_{X}{\left(XX^{⊤}\right)}^{-1}X\varepsilon \varepsilon^{⊤})\right]+\frac{{\sigma^{\prime }}^{2}d}{n}\\ \; & ={\sigma^{\prime }}^{2}E_{X}\left[\mathrm{Tr}({\left(XX^{⊤}\right)}^{-1}\Sigma_{X})\right]+\frac{{\sigma^{\prime }}^{2}d}{n}. \end{array}$$

Note that $X_{i}$’s are i.i.d. samples from $N(0,\Sigma_{X})$, then we have ${\left(XX^{⊤}\right)}^{-1}$ distributed according to ${\mathrm{Wishart}}^{-1}\left(\Sigma_{X}^{-1},n\right)$, where ${\mathrm{Wishart}}^{-1}$ denotes the inverse Wishart distribution with *n* degrees of freedom, and $E\left[{\left(XX^{⊤}\right)}^{-1}\right]=\frac{\Sigma_{X}^{-1}}{n-d-1}$. It then follows that

(A3) $$\mathrm{gen}\left(\mu ,P_{W|S}\right)=\frac{{\sigma^{\prime }}^{2}}{n-d-1}\left[\mathrm{Tr}(\Sigma_{X}^{-1}\Sigma_{X})\right]+\frac{{\sigma^{\prime }}^{2}d}{n}=\frac{{\sigma^{\prime }}^{2}d}{n}\left(2+\frac{d+1}{n-d-1}\right).$$

## Appendix B. Proof of Proposition 1

For all $\hat{w}\in \hat{W}$, it can be shown that

(A4) $$\ell \left(\hat{w},\tilde{Z}\right)={\left(\tilde{Y}-{\tilde{X}}^{⊤}\hat{w}\right)}^{2}={\left({\tilde{X}}^{⊤}\left(w^{*}-\hat{w}\right)+\tilde{\varepsilon }\right)}^{2}.$$

Since $\tilde{X}∼N\left(0,\Sigma_{X}\right)$ and $\tilde{\varepsilon }∼N\left(0,{\sigma^{\prime }}^{2}\right)$, then $\ell \left(\hat{w},\tilde{Z}\right)∼\sigma_{\ell }^{2}\chi_{1}^{2}$, where

$$\sigma_{\ell }^{2}≜{\left(\hat{w}-w^{*}\right)}^{⊤}\Sigma_{X}\left(\hat{w}-w^{*}\right)+{\sigma^{\prime }}^{2},$$

and $\chi_{1}^{2}$ denotes the chi-squared distribution with one degree of freedom. Then, the CGF of $\ell (\hat{w},\tilde{Z})$ is

(A5) $$\Lambda_{\ell (\hat{w},\tilde{Z})}\left(\lambda \right)=-\sigma_{\ell }^{2}\lambda -\frac{1}{2}\ln\left(1-2\sigma_{\ell }^{2}\lambda \right),\;\lambda \in \left(-\infty ,\frac{1}{2\sigma_{\ell }^{2}}\right).$$

Thus, $\ell (\hat{w},\tilde{Z})$ is not sub-Gaussian for all λ∈R. However, it can be shown that

(A6) $$\Lambda_{\ell (\hat{w},\tilde{Z})}\left(\lambda \right)\leq \sigma_{\ell }^{4}\lambda^{2},\;\lambda <0.$$

We need the following lemma from the Theorem 1 of [43] to proceed our analysis.

**Lemma** **A1** ([43])**.** *Assume that for all $\hat{w}\in \hat{W}$, $\Lambda_{\ell (\hat{w},\tilde{Z})}\left(\lambda \right)\leq \frac{\sigma^{2}\lambda^{2}}{2}$ for λ≤0. Then,*

(A7) $$\begin{array}{cc} \mathrm{gen}(\mu ,P_{\hat{W}|S}) & \leq \sqrt{\frac{2\sigma^{2}}{n}I\left(\hat{W};S\right)}. \end{array}$$

Recall that $C\left(w^{*}\right)=\sup_{\hat{w}\in \hat{W}}{∥\hat{w}-w^{*}∥}_{2}^{2}$. We then have the following bound on the CGF of $\ell (\hat{w},\tilde{Z})$,

(A8) $$\Lambda_{\ell (\hat{w},\tilde{Z})}\left(\lambda \right)\leq \lambda^{2}\max_{\hat{w}\in \hat{W}}\sigma_{\ell }^{4}\leq \lambda^{2}{\left(C\left(w^{*}\right)∥\Sigma_{X}∥+{\sigma^{\prime }}^{2}\right)}^{2},\;\lambda <0.$$

Applying Lemma A1 and data processing inequality, we have

(A9) $$\begin{array}{c} \mathrm{gen}\left(\mu ,P_{\hat{W}|S}\right)\leq 2\left(C\left(w^{*}\right)∥\Sigma_{X}∥+{\sigma^{\prime }}^{2}\right)\sqrt{\frac{I(\hat{W};W)}{n}}. \end{array}$$

## Appendix C. Proof of Proposition 2

The constraint on the distortion function can be written as follows:

(A10) $$\begin{array}{cc} D & \geq E_{S,W,\hat{W}}\left[d_{S}\left(\hat{W},W\right)\right]=\frac{1}{n}E_{S,W,\hat{W}}\left[{\left(\hat{W}-W\right)}^{⊤}XX^{⊤}\left(\hat{W}-W\right)\right]. \end{array}$$

It follows from Lemma 1 that

(A11) $$\begin{array}{c} R\left(D\right)=\min_{P_{\hat{W}|W}}I\left(\hat{W};W\right),\;s.t.\;E_{S,W,\hat{W}}\left[{\left(\hat{W}-W\right)}^{⊤}\frac{1}{n}XX^{⊤}\left(\hat{W}-W\right)\right]\leq D. \end{array}$$

Note that $E\left[W\right]=w^{*}$ and $\mathrm{Cov}\left[W\right]=\frac{{\sigma^{\prime }}^{2}}{n-d-1}\Sigma_{X}^{-1}$ since *W* is the ERM solution. In the following proof, we consider a Gaussian random vector with the same mean and covariance matrix $W_{G}∼N\left(w^{*},\frac{{\sigma^{\prime }}^{2}}{n-d-1}\Sigma_{X}^{-1}\right)$ as *W*.

For the upper bound of R(D), consider the channel $P_{\hat{W}|W}^{*}=N\left(\left(1-\alpha \right)W+\alpha w^{*},\left(1-\alpha \right)\frac{D}{d}\Sigma_{X}^{-1}\right)$, where $\alpha =\frac{nD}{d{\sigma^{\prime }}^{2}}\leq 1$. It can be verified that this channel satisfies the constraint on the distortion:

(A12) $$\begin{array}{cc} \; & E_{S,W,\hat{W}}\left[d_{S}\left(\hat{W},W\right)\right]\\ \; & =\alpha^{2}E\left[{\left(W-w^{*}\right)}^{⊤}\frac{1}{n}XX^{⊤}\left(W-w^{*}\right)\right]+\left(1-\alpha \right)\frac{D}{d}\mathrm{Tr}\left(E\left[\frac{1}{n}XX^{⊤}\right]\Sigma_{X}^{-1}\right)\\ \; & =\alpha^{2}E\left[{\left({\left(XX^{⊤}\right)}^{-1}X\varepsilon \right)}^{⊤}\frac{1}{n}XX^{⊤}\left({\left(XX^{⊤}\right)}^{-1}X\varepsilon \right)\right]+\left(1-\alpha \right)D\\ \; & =\alpha^{2}\frac{1}{n}E\left[\varepsilon^{⊤}X^{⊤}{\left(XX^{⊤}\right)}^{-1}X\varepsilon \right]+\left(1-\alpha \right)D\\ \; & =D. \end{array}$$

If we let $\xi ∼N(0,\left(1-\alpha \right)\frac{D}{d}\Sigma_{X}^{-1})$, it follows that

(A13) $$\begin{array}{cc} R(D) & \leq I(W;\left(1-\alpha \right)W+\alpha w^{*}+\xi )\\ \; & \overset{\left(a\right)}{\leq }I\left(W_{G};\left(1-\alpha \right)W_{G}+\xi \right)\\ \; & =\frac{d}{2}\ln\left(\frac{d{\sigma^{\prime }}^{2}}{(n-d-1)D}-\frac{n}{n-d-1}+1\right)\\ \; & \leq \frac{d}{2}{\left(\ln\frac{d{\sigma^{\prime }}^{2}}{(n-d-1)D}\right)}^{+}, \end{array}$$

where (a) is due to the fact that Gaussian distribution maximizes the mutual information in an additive white Gaussian noise channels.

The upper bound on D(R) follows immediately from the upper bound on R(D).

## Appendix D. Discussion of Remark 2

Suppose that $\frac{1}{n}XX^{⊤}$ can be approximated by $\Sigma_{X}$ for large *n* in (A10). It then follows that

(A14) $$\begin{array}{c} R\left(D\right)=\min_{P_{\hat{W}|W}}I\left(\hat{W};W\right),\;s.t.\;E_{S,W,\hat{W}}\left[{\left(\hat{W}-W\right)}^{⊤}\Sigma_{X}\left(\hat{W}-W\right)\right]\leq D. \end{array}$$

It can be easily verified that the channel $P_{W|\hat{W}}^{*}=N\left(\hat{W},\frac{D}{d}\Sigma_{X}^{-1}\right)$ satisfies the distortion constraint. For any $P_{W|\hat{W}}$ such that $E_{S,W,\hat{W}}\left[d_{S}\left(\hat{W},W\right)\right]\leq D$, it follows that

(A15) $$\begin{array}{cc} I(W;\hat{W}) & =E_{W,\hat{W}}\left[\ln\frac{P_{W|\hat{W}}}{P_{W}}\right]\\ \; & =E_{W,\hat{W}}\left[\ln\frac{P_{W|\hat{W}}}{P_{W|\hat{W}}^{*}}\right]+E_{W,\hat{W}}\left[\ln\frac{P_{W|\hat{W}}^{*}}{P_{W_{G}}}\right]-\mathrm{KL}\left(P_{W}∥P_{W_{G}}\right)\\ \; & \geq E_{W,\hat{W}}\left[\ln\frac{P_{W|\hat{W}}^{*}}{P_{W_{G}}}\right]-\mathrm{KL}\left(P_{W}∥P_{W_{G}}\right), \end{array}$$

where $\mathrm{KL}(P_{W}∥P_{W_{G}})$ is the Kullback–Leibler divergence between the two distributions, and the last step follows from the fact that $\mathrm{KL}(P_{W,\hat{W}}∥P_{W,\hat{W}}^{*})\geq 0$. Note that

(A16) $$\begin{array}{cc} \; & E_{W,\hat{W}}\left[\ln\frac{P_{W|\hat{W}}^{*}}{P_{W_{G}}}\right]\\ \; & =E_{W,\hat{W}}\left[\frac{\left(n-d-1\right){\left(W-w^{*}\right)}^{⊤}\Sigma_{X}\left(W-w^{*}\right)}{2{\sigma^{\prime }}^{2}}-\frac{d{\left(\hat{W}-W\right)}^{⊤}\Sigma_{X}\left(\hat{W}-W\right)}{2D}\right]\\ \; & \;+\frac{d}{2}\ln\frac{d{\sigma^{\prime }}^{2}}{(n-d-1)D}\\ \; & \overset{\left(a\right)}{=}\frac{d}{2}\ln\frac{d{\sigma^{\prime }}^{2}}{(n-d-1)D}+E_{W,\hat{W}}\left[\frac{d}{2}-\frac{d{\left(\hat{W}-W\right)}^{⊤}\Sigma_{X}\left(\hat{W}-W\right)}{2D}\right]\\ \; & \overset{\left(b\right)}{\geq }\frac{d}{2}\ln\frac{d{\sigma^{\prime }}^{2}}{(n-d-1)D}, \end{array}$$

where (a) follows from the fact that $E\left[W\right]=w^{*}$ and $\mathrm{Cov}\left[W\right]=\frac{{\sigma^{\prime }}^{2}}{n-d-1}\Sigma_{X}^{-1}$, and (b) is due to the fact that $P_{\hat{W}|W}$ satisfies the distortion constraint. Thus,

(A17) $$R\left(D\right)\geq \frac{d}{2}\ln\frac{d{\sigma^{\prime }}^{2}}{(n-d-1)D}-\mathrm{KL}\left(P_{W}∥P_{W_{G}}\right).$$

References yes
